# COVID-19 and non-communicable diseases

**DOI:** 10.1136/postgradmedj-2020-137742

**Published:** 2020-03-30

**Authors:** Rimesh Pal, Sanjay K Bhadada

**Affiliations:** Endocrinology, Post Graduate Institute of Medical Education and Research, Chandigarh, India; Endocrinology, Post Graduate Institute of Medical Education and Research, Chandigarh, India

**Keywords:** infectious diseases

Since December 2019, a novel coronavirus disease (COVID-19), caused by severe acute respiratory syndrome coronavirus 2 (SARS-CoV-2), has scourged the world, resulting in the WHO declaring it as a pandemic. As of 19 March 2020, COVID-19 has affected over 207 000 people in at least 166 countries worldwide, with most of the cases being reported from China and Europe. The absolute number of deaths has already surpassed 8500 globally and is expected to increase further as the disease spreads rapidly. The disease has also infiltrated the Indian masses and is spreading fast.

Although the overall fatality rate of COVID-19 is low,^1^ older adults and patients with comorbidities are more likely to have severe disease and subsequent mortality. The most commonly reported non-communicable diseases that have been shown to predict poor prognosis in patients with COVID-19 include diabetes mellitus (DM), hypertension, cerebrovascular disease, coronary artery disease (CAD) and chronic obstructive pulmonary disease (COPD).^1–4^ Wang *et al*^4^ reported that among the 26.1% of patients shifted to intensive care unit (ICU), 72.2% had concurrent comorbidities, as opposed to only 37.3% of patients who did not require ICU care. Yang and colleagues^3^ had shown that among the 32 non-survivors from a group of 52 ICU patients, the most distinctive pre-existing non-communicable comorbidities were cerebrovascular diseases (22%) and diabetes (22%). Of the 1099 confirmed patients with COVID-19 reported by Guan *et al*^1^, 173 had severe disease; as compared with those with non-severe disease, patients with severe disease had a higher prevalence of hypertension (13.4% vs 23.75%), DM (5.7% vs 16.2%), CAD (1.8% vs 5.8%), COPD (0.6% vs 3.5%) and cerebrovascular disease (1.2% vs 2.3%). A recently conducted meta-analysis concluded that hypertension, respiratory system disease and cardiovascular disease had an OR of 2.36, 2.46 and 3.42, respectively, for severe disease as compared with non-severe disease.^2^

Underlying comorbidities have been poor predictors of outcome in other viral infections as well. Hong *et al*^5^ had reported that in patients with seasonal influenza, DM and chronic cardiovascular disease were significantly associated with the development of complications. Although it can be assumed that patients with comorbidities are more likely to be older than those without them, and in fact most of the aforementioned studies did not adjust for age, multiple additional explanations can be put forward for this apparent association between pre-existing non-communicable diseases and COVID-19 severity. Patients with DM and COPD have underlying immunodeficiency, which may make them more susceptible to COVID-19 complications. Innate immunity, the first line of defence against SARS-CoV-2, is inevitably compromised in patients with uncontrolled DM, thereby allowing unhindered proliferation of the pathogen within the host. In addition, exaggerated cytokine response in the absence of an immunostimulation is characteristic of DM^6^; this might contribute to hyperproduction of proinflammatory cytokines, notably interleukin (IL)-1, IL-6 and tumour necrosis factor-α, seen in patients with COVID-19 complicated by acute respiratory distress syndrome. Likewise, inactivation of the innate immune system and underexpression of pulmonary interferon-β, a cytokine involved in the defence against coronavirus, are observed in patients with COPD.^7^

The connecting link between coexistence of hypertension and atherosclerotic disease (CAD or cerebrovascular disease) with COVID-19 severity is possibly ACE2. ACE2 is a type 1 integral membrane glycoprotein that is constitutively expressed by the epithelial cells of the lungs, kidney, intestine and blood vessels. SARS-CoV-2 uses ACE2 as a receptor for entry into the host pneumocytes.^8^ Hence, any condition that upregulates ACE2 is likely to facilitate infection with COVID-19. However, contrary to what one would anticipate, overall ACE2 expression is reduced in patients with diabetes, hypertension and atherosclerosis.^9^ Herein comes the confounding role of ACE inhibitors (ACEi) and angiotensin receptor blockers (ARBs), drugs that are so widely used in patients with DM, hypertension and cardiovascular disease. The expression of ACE2 is markedly increased in patients with DM and hypertension being treated with ACEi or ARBs.^10^ Thus, use of ACE2-stimulating drugs might be the underlying cause of severe and fatal COVID-19 disease seen in patients with metabolic comorbidities. Unfortunately, none of the recently conducted studies has taken into account the baseline treatment.

Whatever may be the underlying pathogenic mechanisms, community-dwelling subjects harbouring underlying non-communicable diseases are definitely at an increased risk of severe and fatal COVID-19 disease. India is home to over 1.3 billion people with diabetes and hypertension prevalence of 7.3% and 28.9%, respectively. Considering such large numbers, it is imperative that community-dwellers with underlying comorbidities take extra precautions not to contract the virus. Social distancing and strict hand and respiratory hygiene are the need of the hour. People with DM should ensure good glucose control as improvement in glycaemia does boost the host immune response. Although organisations all over the world recommend the continuation of ACEi/ARBs in patients with diabetes and hypertension largely because of lack of robust data to support their cessation, we believe that calcium channel blockers might be a viable alternative, as they do not upregulate ACE2 levels.
